# Quantitative Trace Analysis of Dilute Mixtures Using a Benchtop NMR System with SABRE Hyperpolarization

**DOI:** 10.1021/acs.analchem.5c01026

**Published:** 2025-05-19

**Authors:** Bono O. Jimmink, Mattia Negroni, Thom B. Posthumus, Arno P. M. Kentgens, Marco Tessari

**Affiliations:** Magnetic Resonance Research Center, Institute for Molecules and Materials, 6029 Radboud University, 6525AJ Nijmegen, The Netherlands

## Abstract

Despite their modest sensitivity, benchtop NMR spectrometers have recently attracted a great deal of attention, because of their low cost, high portability, and robustness. A solution to the sensitivity limitation of benchtop spectrometers is offered by nuclear spin hyperpolarization, by which large NMR signal enhancements can be realized. Signal Amplification By Reversible Exchange (SABRE) is one-such hyperpolarization technique, which utilizes hydrogen enriched in the *para* spin-isomer (*p*H_2_). However, the application of SABRE with benchtop NMR has so far largely been restricted to sample concentrations in the millimolar range. In this work, we present SABRE hyperpolarization of a mixture at micromolar concentrations, measured on a 1 T benchtop spectrometer. The observed linear dependence between hyperpolarized signals and concentration demonstrates the stability of our approach, which allows quantification in the micromolar range.

## Graphical abstract

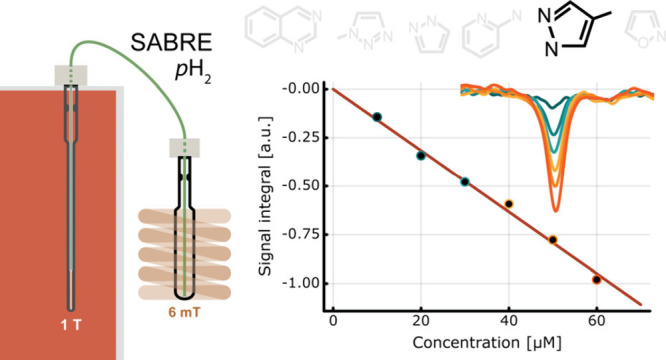

In the past decade, cryogen-free compact spectrometers, commonly called benchtop spectrometers, have become increasingly popular in the field of nuclear magnetic resonance (NMR) spectroscopy. These spectrometers typically operate at moderate fields of 43–100 MHz (^1^H resonance frequency) and are more portable and inexpensive than conventional NMR spectrometers.^1^ It has been demonstrated that such devices are capable of accurate quantitative analysis and have already been applied, for example, to the study of cannabinoids,^2^ microplastics,^3^ lignin,^4^ wines,^5^ and reaction monitoring.^6^ However, due to the low magnetic field, the sensitivity of benchtop instruments is significantly reduced compared to that of high-field NMR spectrometers, with concentration requirements typically in the medium-high millimolar range. Nuclear spin hyperpolarization^7^ has been employed on several occasions in combination with benchtop spectrometers to increase NMR sensitivity. Particularly, Signal Amplification By Reversible Exchange (SABRE)^8,9^ can provide a low-cost and easy-to-implement route to NMR sensitivity enhancement for benchtop instruments.^10–15^

SABRE occurs spontaneously at low field,^16^ as *para*-enriched hydrogen and substrate molecules weakly associate with an iridium complex, causing the conversion of dihydride singlet order into enhanced magnetization of the substrate (Figure 1).

**Figure 1:**
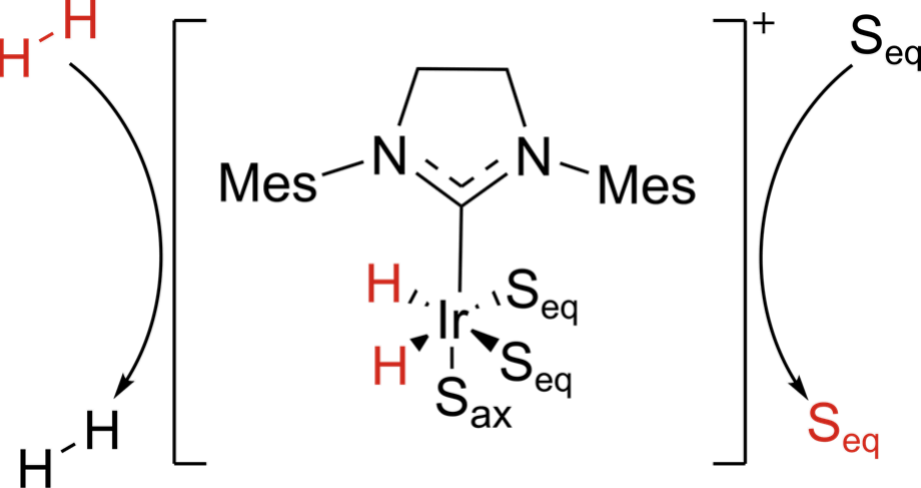
Representation of SABRE hyperpolarization. The *p*H_2_ and substrate (S) molecule transiently bind to the iridium-carbene SABRE catalyst^17,18^ and, at 6 mT, the nonequilibrium spin order (red) is transferred spontaneously between the two.

Within the context of benchtop NMR, SABRE has primarily been applied at millimolar concentration on solutions of pure compounds.^19,20^ Although the extension of SABRE to complex mixtures of dilute analytes has been previously demonstrated by Eshuis et al. for high-field instruments,^21^ the NMR community has so far been rather reluctant to implement these conditions at low field. In this paper, we demonstrate that Eshuis’s approach can be ported to benchtop NMR to measure mixtures in the low micromolar regime. While SABRE hyperpolarization is expected to perform equally well, NMR acquisition at 1 T with a room-temperature insert determines a ca. 20-fold sensitivity drop, compared to a high field instrument employing a cryo-cooled probe. In order to achieve a suitable signal-to-noise ratio at micromolar concentration, SABRE hyperpolarization was then combined with extensive signal averaging. This required a system for automated shuttling of the sample between a vessel at 6 mT–where *para*-enriched hydrogen is bubbled through the solution—and the benchtop spectrometer for NMR acquisition. The stability and robustness of our approach are witnessed by the linear dependence of signal integral versus concentration measured in a series of samples for one of the analytes in the mixture.

This work illustrates how the application of SABRE can extend the use of benchtop NMR to low concentration, toward, for example, the trace analysis of natural extracts or biofluids.

## Results and Discussion

### Deuterated Pyridine as a Cosubstrate

As previously demonstrated,^22^ SABRE efficiency at low analyte concentration can be restored by adding an excess of 1-methyl-1,2,3-triazole ligand (referred to as a “cosubstrate”) to the solution. However, this choice of cosubstrate at low fields would result in large signals dominating the aromatic region, which would hamper the analysis of the NMR spectrum. Alternatively, deuterated molecules, such as pyridine-*d*_5_ may prove an excellent choice of cosubstrate, because they have been shown to yield higher analyte enhancements.^23^ Daniele et al. showed that deuterated pyridine can be effectively used as a cosubstrate.^24^ However, signals corresponding to the *ortho*-protons of pyridine could still be observed in the acquired spectra, which was attributed to the well-known *ortho*-directed hydrogen isotope exchange of iridium catalysts.^18,25,26^ Although pyridine-*d*_5_ can be successfully used as a cosubstrate for short experiments, the continued isotope exchange^11,26^ hampers longer multiscan experiments due to the—eventually—large pyridine *ortho*-proton signal, and associated instabilities.

This issue was partly addressed by using [Ir­(SIMes)­(COD)]Cl as a catalyst precursor,^18^ rather than the more widely utilized [Ir­(IMes)­(COD)]­Cl, which resulted in a significant decrease in the intensity of the pyridine *ortho*-proton signal. This is illustrated in Figure S1, where the signal integral of SABRE-hyperpolarised *ortho*-protons of pyridine is plotted as a function of time, for similar solutions of pyridine-*d*_5_, using either catalyst at the same conditions. Remarkably, where the sample using [Ir­(IMes)­(COD)]Cl displays strong signals, the sample with [Ir­(SIMes)­(COD)]­Cl shows a near-nothing signal integral. This unlocks the possibility of using pyridine-*d*_5_ for multiscan experiments minimizing the interference from a continuously increasing pyridine signal, as is shown in the next section.

### Hyperpolarization of a Mixture

A mixture of six components, each at a concentration of 40 μM, was hyperpolarized using SABRE. In addition to the six dilute compounds, the SABRE mixture consisted of 100 μM [Ir­(SIMes)­(COD)]Cl catalyst precursor and 1 mM of pyridine-*d*_5_, which acts as a cosubstrate. The employed SABRE setup (Figure S2) allows for automated shuttling of the sample between a vessel at 6 mT—where *para*-enriched hydrogen is bubbled through the solution—and the benchtop spectrometer. Such an automated system allows the acquisition of multiple consecutive scans to improve the signal-to-noise ratio; in some instances, similar devices have also been used to perform two-dimensional (2D) experiments.^10,27^ After a brief activation time, repeated scans show a good stability, with a standard deviation of approximately 8% for the hyperpolarized signals, as estimated from the methyl signal integral of 1-methyl-1,2,3-triazole over an array of 16 spectra, each acquired with two transients. In this manner, signal averaging and phase cycling over multiple scans is possible. This is in stark contrast to the more common manual shake-and-insertion method of SABRE, which has been reported to have ∼20% variability,^28^ and slightly above the deviation reported for other systems.^10,29^ A representative spectrum of a sample with the above-mentioned composition, recorded with 32 scans, is shown in Figure 2. Signal-to-noise ratios, ranging between 20 and 135, were obtained in the experiment. Please note that, in addition to the six dilute compounds, a signal corresponding to *ortho*-protons of pyridine is present in the spectrum, originating from hydrogen isotope exchange as previously discussed. Samples at low-micromolar concentration could not be measured under thermal conditions; therefore, a reference sample of 10 mM 3-methylpyrazole was used to estimate signal enhancements on the order of 750. This value is comparable to the one reported at higher concentrations for automated SABRE systems in benchtop NMR.^10,29,30^ Such signal enhancements in the micromolar regime put benchtop NMR on par with modern high-field NMR spectrometers.

**Figure 2:**
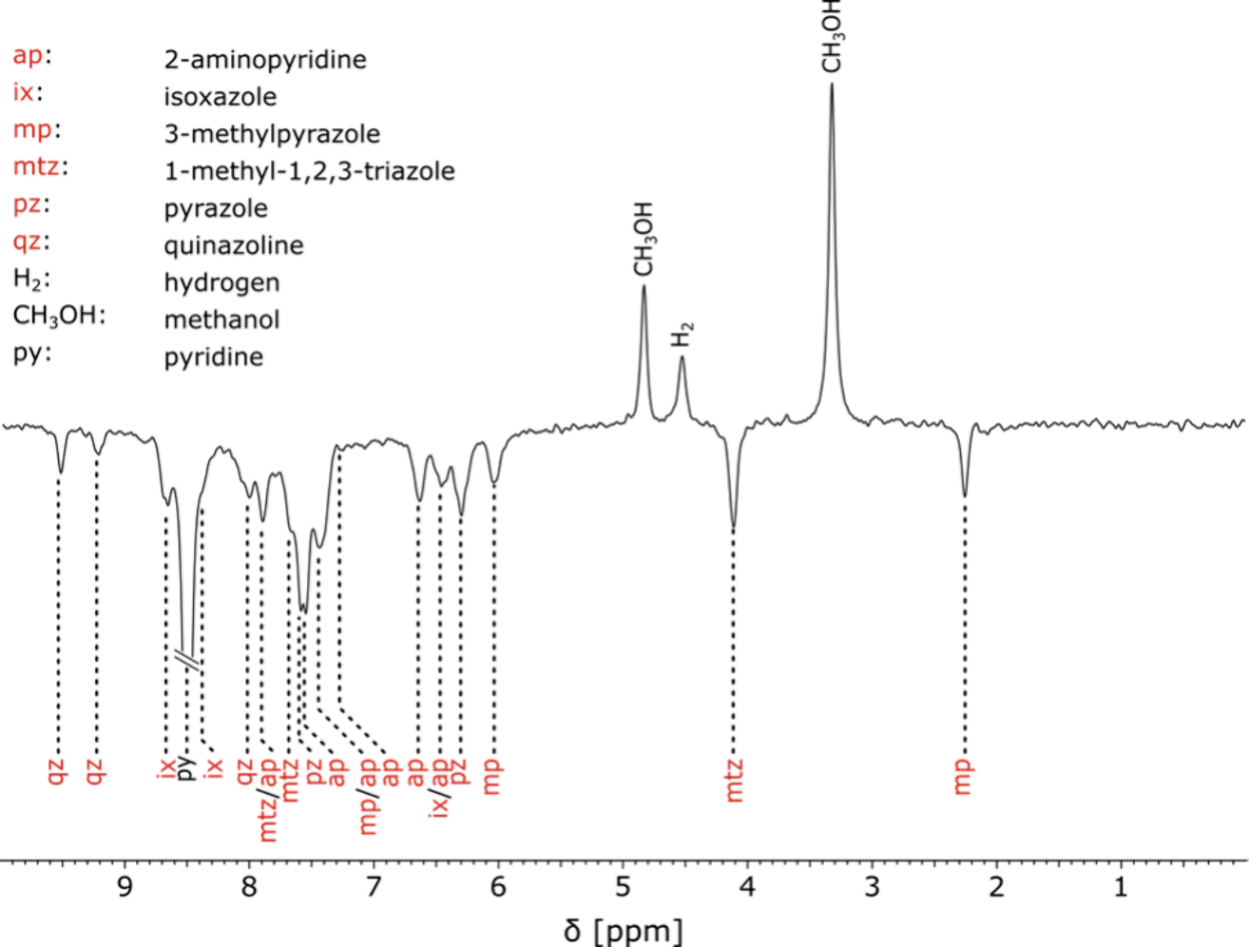
^1^H NMR spectrum acquired at 43 MHz after SABRE hyperpolarization at 6 mT with 51% *para*-enriched H_2_. The sample consisted of a mixture of 1 mM pyridine-*d*_5_, 100 μM [Ir­(SIMes)­(COD)]Cl precatalyst, and 40 μM each of the other analytes, in methanol-*d*_4_.

### Linear Signal Response of 3-Methylpyrazole

SABRE enhancements depend on several molecule-dependent parameters—such as the nuclear spin relaxation rate, scalar coupling constants, and dissociation rates—meaning that quantitative analysis by integral comparison with a standard is unfeasible.^21^ However, Eshuis et al. showed that dilute components in SABRE exhibit a linear dependence between signal and concentration,^22^ which allows analyte quantification by standard addition. In order to demonstrate the stability of our system, six samples of similar composition as detailed above, but with varying concentrations of 3-methylpyrazole in a range of 10–60 μM, were measured under the same conditions. The methyl signal from 3-methylpyrazole could be reliably integrated, as it is well-separated from the others; its integrals, as a function of concentration, are displayed in the plot in Figure 3. Based on this plot, the limit of detection (defined as a signal-to-noise ratio of 5) for 3-methylpyrazole can be estimated to be 5 μM. The uncertainty of the measurement is calculated from the standard deviation of the signal integral of 1-methyl-1,2,3-triazole over the concentration series (9.6%). This variance is consistent with the interscan variance and shows that the SABRE conditions are sufficiently stable. The deviation between the intersample variance in our experiments and the one reported for benchtop NMR at thermal equilibrium (4%)^31,32^ may be attributed to instabilities due to continuous shuttling to and from the spectrometer. The integrated values, as a function of the concentration, exhibit excellent linearity.

**Figure 3:**
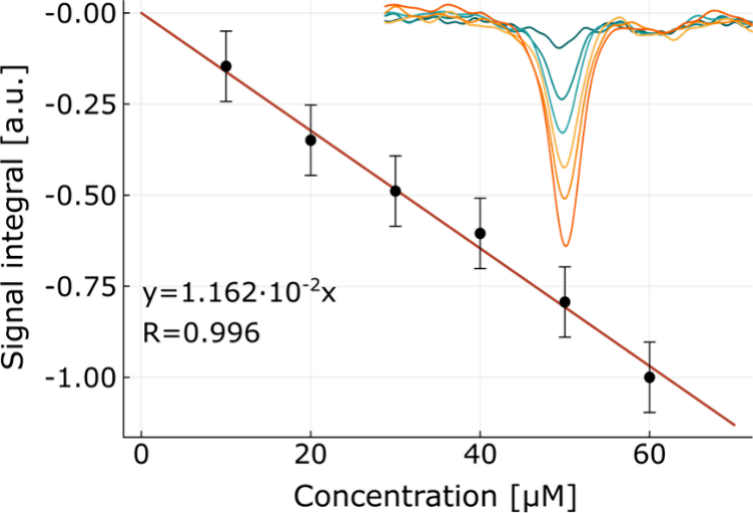
Integral of the SABRE-enhanced ^1^H NMR signal of 3-methylpyrazole at 2.25 ppm (see subplot) as a function of concentration (full spectra are shown in Figure S5). Error bars correspond to the estimated intersample variation (see text).

## Conclusion

In conclusion, we have shown that SABRE hyperpolarization can be an effective tool to overcome the low-sensitivity limitation of benchtop NMR spectrometers. Using relatively low-cost and simple instrumentation, spectra of mixtures in the low micromolar regime were obtained, making this approach competitive with conventional high-field machines, in terms of sensitivity. In addition, the stability of our approach was demonstrated by the linear dependence between the concentration and signal integral. This is important when using the SABRE-benchtop for quantifying low-concentration analytes in mixtures. Extending the current methodology to multidimensional NMR can improve the spectral resolution, which would facilitate the use of benchtop spectrometers for the quantitative trace analysis of natural extracts, biofluids, or reaction (by)­products.

## Supplementary Material



## Data Availability

Raw data and scripts used for NMR processing are available upon request.

## Notes

Materials and methods, *ortho*-pyridine signal as a function of time for the two catalysts, schematic of the SABRE system, chemical structures of mixture components, comparison of various bubbling times, full spectra of concentration series, concentration series in the presence of signal overlap (PDF)

## References

[1] van
Beek T. A. (2021). Low-field benchtop NMR spectroscopy: status and prospects
in natural product analysis. Phytochemical Analysis.

[2] Araneda J.
F., Chu T., Leclerc M. C., Riegel S. D., Spingarn N. (2020). Quantitative analysis
of cannabinoids using benchtop NMR instruments. Analytical Methods.

[3] Peez N., Rinesch T., Kolz J., Imhof W. (2022). Applicable and cost-efficient
microplastic analysis by quantitative 1H-NMR spectroscopy using benchtop
NMR and NoD methods. Magn. Reson. Chem..

[4] Gracia-Vitoria J., Rubens M., Feghali E., Adriaensens P., Vanbroekhoven K., Vendamme R. (2022). Low-field benchtop versus high-field
NMR for routine 31P analysis of lignin, a comparative study. Industrial Crops and Products.

[5] Matviychuk Y., Haycock S., Rutan T., Holland D. J. (2021). Quantitative
analysis
of wine and other fermented beverages with benchtop NMR. Anal. Chim. Acta.

[6] Maschmeyer T., Yunker L. P., Hein J. E. (2022). Quantitative and convenient real-time
reaction monitoring using stopped-flow benchtop NMR. Reaction Chemistry & Engineering.

[7] Eills J., Budker D., Cavagnero S., Chekmenev E. Y., Elliott S. J., Jannin S., Lesage A., Matysik J. r., Meersmann T., Prisner T. (2023). Spin hyperpolarization
in modern magnetic resonance. Chem. Rev..

[8] Adams R. W., Aguilar J. A., Atkinson K. D., Cowley M. J., Elliott P. I., Duckett S. B., Green G. G., Khazal I. G., López-Serrano J., Williamson D. C. (2009). Reversible interactions with para-hydrogen enhance
NMR sensitivity by polarization transfer. Science.

[9] Atkinson K. D., Cowley M. J., Elliott P. I., Duckett S. B., Green G. G., Lopez-Serrano J., Whitwood A. C. (2009). Spontaneous transfer of para hydrogen
derived spin order to pyridine at low magnetic field. J. Am. Chem. Soc..

[10] Richardson P. M., Parrott A. J., Semenova O., Nordon A., Duckett S. B., Halse M. E. (2018). SABRE hyperpolarization
enables high-sensitivity 1
H and 13 C benchtop NMR spectroscopy. Analyst.

[11] Semenova O., Richardson P. M., Parrott A. J., Nordon A., Halse M. E., Duckett S. B. (2019). Reaction
monitoring using SABRE-hyperpolarized benchtop
(1 T) NMR spectroscopy. Analytical chemistry.

[12] Tennant T., Hulme M. C., Robertson T. B., Sutcliffe O. B., Mewis R. E. (2020). Benchtop NMR analysis of piperazine-based drugs hyperpolarised
by SABRE. Magn. Reson. Chem..

[13] Silva
Terra A. I., Rossetto M., Dickson C. L., Peat G., Uhrin D., Halse M. E. (2023). Enhancing ^19^F Benchtop
NMR spectroscopy by combining para-hydrogen hyperpolarization and
multiplet refocusing. ACS Measurement Science
Au.

[14] Alcicek S., Van Dyke E., Xu J., Pustelny S., Barskiy D. A. (2023). 13C and
15N benchtop NMR detection of metabolites via relayed hyperpolarization. Chem.-Methods.

[15] Kircher R., Xu J., Barskiy D. A. (2024). In Situ Hyperpolarization Enables 15N and 13C Benchtop
NMR at Natural Isotopic Abundance. J. Am. Chem.
Soc..

[16] Barskiy D. A., Knecht S., Yurkovskaya A. V., Ivanov K. L. (2019). SABRE: Chemical
kinetics and spin dynamics of the formation of hyperpolarization. Prog. Nucl. Magn. Reson. Spectrosc..

[17] van
Weerdenburg B. J. A., Gloggler S., Eshuis N., Engwerda A. H. J., Smits J. M. M., de Gelder R., Appelt S., Wymenga S. S., Tessari M., Feiters M. C., Blumich B., Rutjes F. P. J. T. (2013). Ligand
effects of NHC–iridium catalysts for signal amplification by
reversible exchange (SABRE). Chem. Commun..

[18] Lloyd L. S., Asghar A., Burns M. J., Charlton A., Coombes S., Cowley M. J., Dear G. J., Duckett S. B., Genov G. R., Green G. G. (2014). Hyperpolarisation
through reversible interactions
with para hydrogen. Catal. Sci. Technol..

[19] Silva
Terra A. I., Taylor D. A., Halse M. E. (2024). Hyperpolarised benchtop
NMR spectroscopy for analytical applications. Prog. Nucl. Magn. Reson. Spectrosc..

[20] Silva
Terra A. I., Halse M. E. (2025). Low-Micromolar Quantification of
Fluorinated Analytes Using Hyperpolarized ^19^F Benchtop
Nuclear Magnetic Resonance. Chemistry-Methods.

[21] Eshuis N., Van Weerdenburg B. J., Feiters M. C., Rutjes F. P., Wijmenga S. S., Tessari M. (2015). Quantitative trace analysis of complex mixtures using
SABRE hyperpolarization. Angew. Chem..

[22] Eshuis N., Hermkens N., van Weerdenburg B. J., Feiters M. C., Rutjes F. P., Wijmenga S. S., Tessari M. (2014). Toward nanomolar detection by NMR
through SABRE hyperpolarization. J. Am. Chem.
Soc..

[23] Dücker E. B., Kuhn L. T., Münnemann K., Griesinger C. (2012). Similarity
of SABRE field dependence in chemically different substrates. Journal of magnetic resonance.

[24] Daniele V., Legrand F. X., Berthault P., Dumez J. N., Huber G. (2015). Single-Scan
Multidimensional NMR Analysis of Mixtures at Sub-Millimolar Concentrations
by using SABRE Hyperpolarization. ChemPhysChem.

[25] Brown J. A., Irvine S., Kennedy A. R., Kerr W. J., Andersson S., Nilsson G. N. (2008). Highly active iridium­(I) complexes for catalytic hydrogen
isotope exchange. Chem. Commun..

[26] Barskiy D. A., Kovtunov K. V., Koptyug I. V., He P., Groome K. A., Best Q. A., Shi F., Goodson B. M., Shchepin R. V., Coffey A. M., Waddell K. W., Chekmenev E. Y. (2014). The feasibility
of formation and kinetics of NMR signal amplification by reversible
exchange (SABRE) at high magnetic field (9.4 T). J. Am. Chem. Soc..

[27] Robinson A. D., Richardson P. M., Halse M. E. (2019). Hyperpolarised 1H–13C benchtop
NMR spectroscopy. Applied Sciences.

[28] Mewis R. E., Atkinson K. D., Cowley M. J., Duckett S. B., Green G. G. R., Green R. A., Highton L. A. R., Kilgour D., Lloyd L. S., Lohman J. A. B., Williamson D. C. (2014). Probing
signal amplification by reversible
exchange using an NMR flow system. Magn. Reson.
Chem..

[29] Ellermann F., Saul P., Hövener J.-B., Pravdivtsev A. N. (2023). Modern
manufacturing enables magnetic field cycling experiments and parahydrogen-induced
hyperpolarization with a benchtop NMR. Anal.
Chem..

[30] Yang J., Xin R., Lehmkuhl S., Korvink J. G., Brandner J. J. (2024). Development of a
fully automated workstation for conducting routine SABRE hyperpolarization. Sci. Rep..

[31] Lee Y., Matviychuk Y., Holland D. J. (2020). Quantitative analysis using external
standards with a benchtop NMR spectrometer. J. Magn. Reson..

[32] Shilliday E. R., Barrow B., Langford D., Ling N. N., Robinson N., Johns M. L. (2022). Quantitative measurement of mono-ethylene glycol (MEG)
content using low-field nuclear magnetic resonance (NMR). Journal of Natural Gas Science and Engineering.

