# Supplementary material for: Quantitative Trace Analysis of Dilute Mixtures Using a Benchtop NMR System with SABRE Hyperpolarization
Source: Anal Chem. 2025 May 19;97(21):10962–5. doi: 10.1021/acs.analchem.5c01026 (PMC12138871; doi:10.1021/acs.analchem.5c01026)
Supplement: Supplementary file 1 [file ac5c01026_si_001.pdf]

SUPPORTING INFORMATION

QUANTITATIVE TRACE ANALYSIS OF DILUTE MIXTURES IN A BENCHTOP NMR SYSTEM USING SABRE HYPERPOLARISATION

Bono O. Jimmink,[‡] Mattia Negroni,[‡] Thom B. Posthumus, Arno P.M. Kentgens, Marco Tessari*

TABLE OF CONTENTS

Materials and methods	S-2
Figure S1 Integrated *ortho*-proton signal of pyridine as a function of time for the two catalysts	S-3
Figure S2 SABRE system	S-3
Figure S3 Molecules used in the mixture	S-4
Figure S4 S/N ratio as a function of bubbling time	S-4
Figure S5 SABRE-enhanced ^{1}H NMR spectra of the mixture at different concentration of 3-methylpyrazole	S-5
Figure S6 Linear dependence of signal of 3-methylpyrazole *vs* concentration in the presence of overlap	S-6
References	S-6

MATERIALS AND METHODS

Chemicals. 2-aminopyridine (CAS: 504-29-0), isoxazole (CAS: 288-14-2) were purchased from Across Organics (Thermo Scientific Chemicals), methanol (CAS: 67-56-1) was purchased from Fischer Scientific, 3-methylpyrazole (CAS:1453-58-3), 1-methyl-1,2,3-triazole (CAS:16681-65-5), pyrazole (CAS: 288-13-1), quinazoline (CAS: 253-82-7), methanol-d_4 (CAS: 811-98-3), 1,3-Bis(2,4,6-trimethylphenyl)-4,5-dihydroimidazol-2-ylidene (SIMes) (CAS:173035-11-5) and 1,3-Bis(2,4,6-trimethylphenyl)-1,3-dihydro-2*H*-imidazol-2-ylidene (IMes) (CAS: 141556-42-5) were purchased from Sigma Aldrich and pyridine-d_5 was purchased from Deutero GmbH,. All chemicals were 95% pure or higher and were used without further purification. The pre-catalysts [Ir(SIMes)(COD)]Cl and [Ir(IMes)(COD)]Cl were synthesised according to the procedure described by Kelly III et al.[1] A parahydrogen generator (HyperSpin Scientific) cooled by liquid nitrogen to 77 K was used to convert thermal hydrogen (purity 5.0, Linde Gas Benelux B.V.) to 51% para-enriched hydrogen. Nitrogen gas was obtained from the on-site bulk nitrogen supply (Linde).

SABRE hardware. Where other SABRE systems for benchtop NMR rely on mechanical shuttling[2, 3] or flow systems,[4] we opted for a simpler setup (Fig. S2) with two main containers: the bubbling vessel and the detection tube. The first is placed inside a solenoid able to generate a field of 6 mT needed for SABRE while the latter is kept in the spectrometer. An Arduino MEGA 2560 controls a series of solenoids valves that regulate sample transfer between containers in addition to hydrogen and nitrogen bubbling; the microcontroller is also responsible for signaling the spectrometer to start the pulse sequence. All the solenoid valves are NResearch Inc. 161PK011HP. The transfer is obtained by regulating the pressure between the vessels. A one-way valve on the main gas line prevents the sample from flowing into the valves. A second one-way valve set to 20 psi (~1.38 bar) cracking pressure on the exhaust lines allows the system to stay above atmospheric pressure.

The sample is inserted into the bubbling vessel and subjected to 2 minutes of nitrogen bubbling to remove oxygen from solution. It is then transferred into the detection area for shimming before starting the measure. During measurement the sample is subjected to 2 s of pH2 bubbling and then transferred into the detection area in 3 s. Before triggering the instrument, the sample is left 0.5 s to settle. The sample is then transferred back into the bubbling vessel; a full cycle takes 15 s.

Samples for SABRE hyperpolarisation. SABRE mixtures were prepared from stock solutions of individual components and catalyst precursor in methanol-d_4. SABRE experiments were typically performed with 40 µM of each of analyte, 1 mM pyridine-d_5 and 100 µM of pre-catalyst. 2 µl CH_3OH was added to every solution to facilitate shimming. The solution was transferred to a custom glass 6 ml tube, fitted with an in-house developed headpiece. The sample was purged with nitrogen gas for 2 minutes before being pneumatically shuttled into a quick pressure valve NMR tube (Wilmad 528-QPV-8), which was also sealed with an in-house developed headpiece. Shuttling is performed by creating a difference in pressure between the two containers, using *para*-enriched hydrogen gas. The sample was shimmed before the measurement and thereafter, the SABRE procedure was started. The SABRE procedure consists of shutting the samples back into the 6 ml tube – which is kept at 6 mT – bubbling *para*-enriched hydrogen through the solution for 2 s and then transferring the solution into the benchtop spectrometer in 3 s. The bubbling time has been chosen to give the optimal signal to noise ratio per unit time, while minimising evaporation of the sample (Figure S4). A full cycle of bubbling, shuttling, NMR experiment and reverse-shuttling took 15 s.

NMR. All experiments were performed on a Magritek Spinsolve benchtop spectrometer, operating at 43 MHz ^{1}H resonance frequency. A 1D one-pulse experiment was performed using a 90-degree pulse and acquiring an FID of 4096 points in 819.2 ms. Each experiment was recorded with 8 dummy scans and 32 scans, using a two-step (ϕ_{rf}=0°,90°; ϕ_{rec}=0°,90°) phase cycle. All spectra were processed using NMRPipe,[5] applying a Lorentz-to-Gauss apodisation function, zero-filling to 65k points, Fourier transformation and polynomial baseline correction. Reference deconvolution using the FIDDLE algorithm[6] was employed to reduce shimming imperfections.

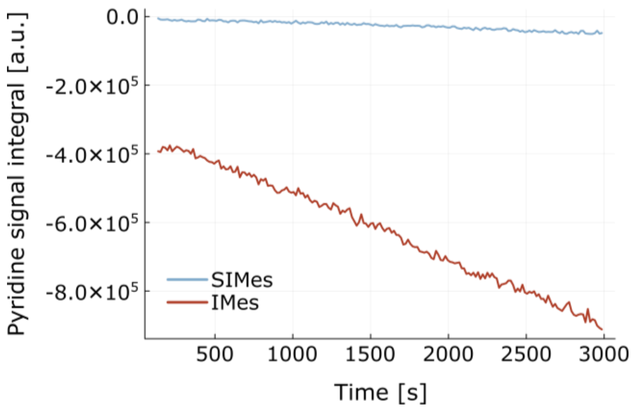

Figure S1. Integrated *ortho*-proton signal of pyridine as a function of time. The samples consisted of 2 µl CH_3OH 1 mM pyridine-d_5 and 100 µM [Ir(SIMes)(COD)]Cl or [Ir(IMes)(COD)]Cl in methanol-d_4. The samples are labeled respectively as SIMes (blue) and IMes (red) in the figure legend.

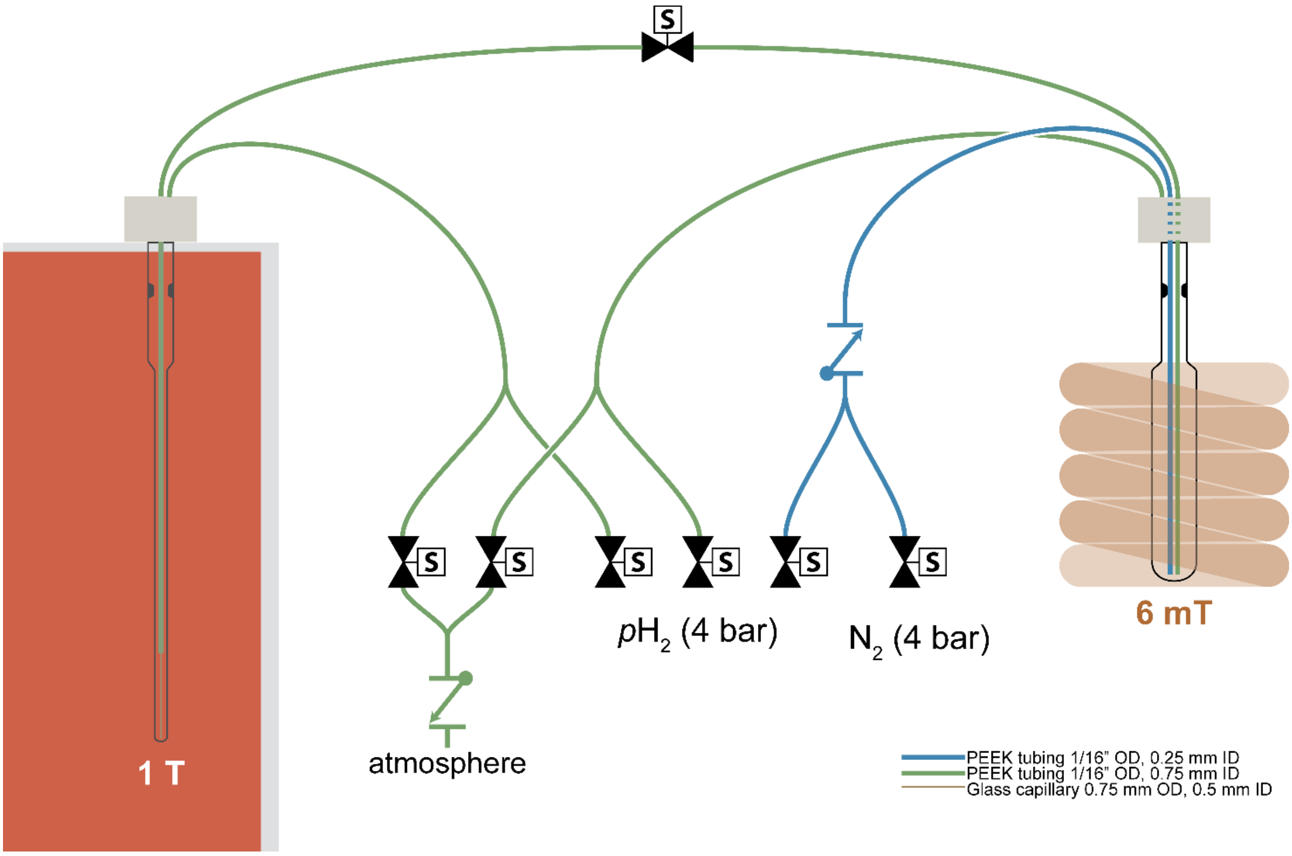

Figure S2. Schematic representation of the SABRE system.

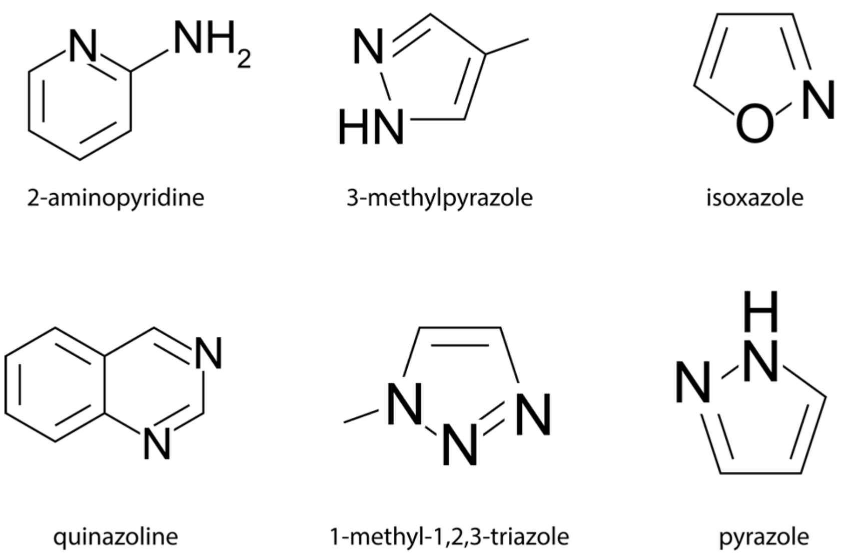

Figure S3. Molecules used in the mixture.

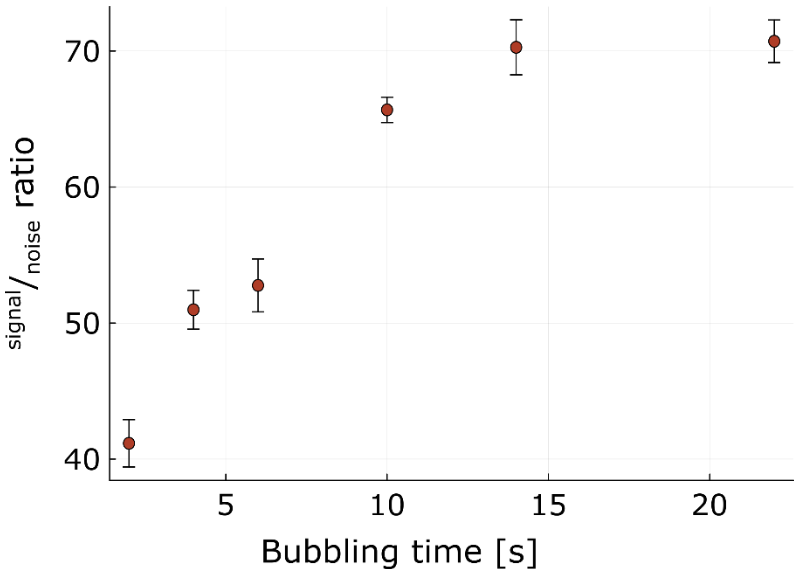

Figure S4. S/N ratio as a function of bubbling time.

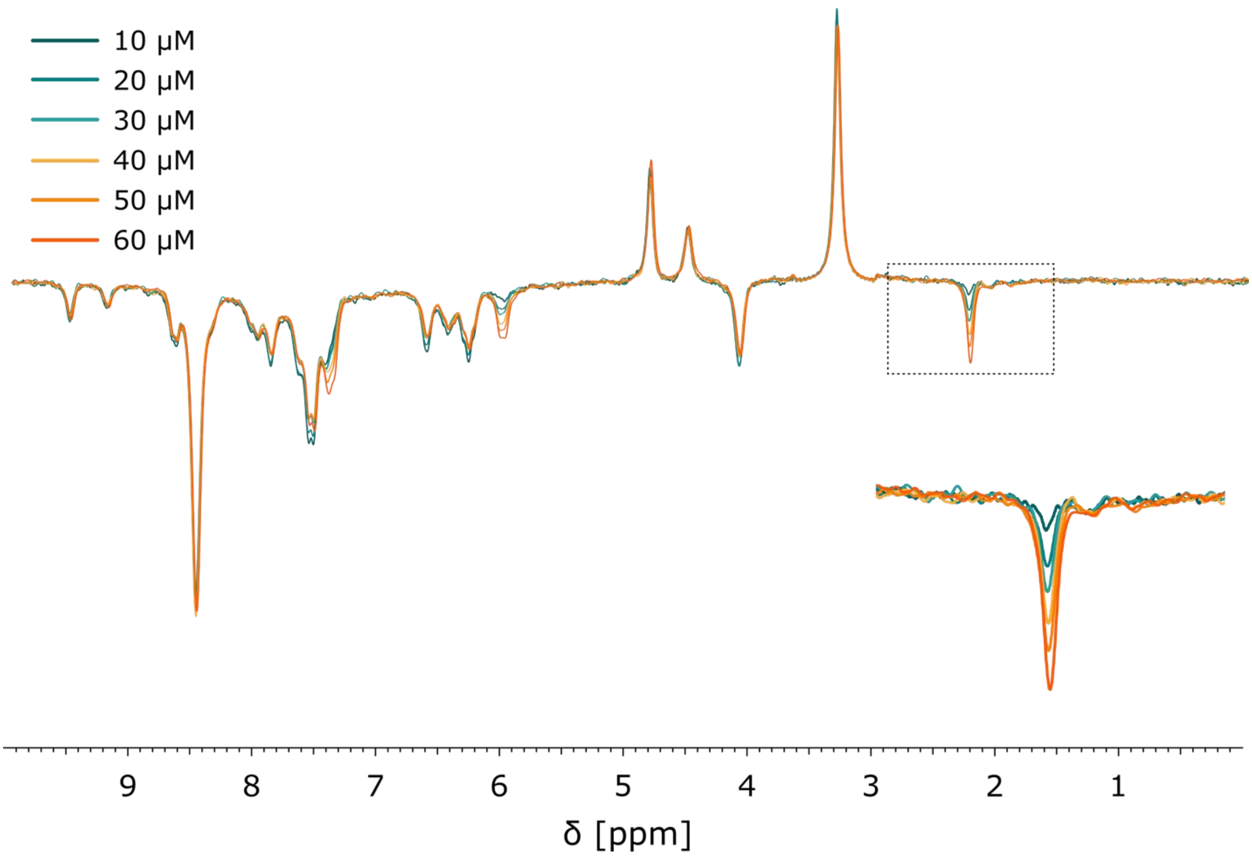

Figure S5. SABRE-enhanced ^{1}H NMR spectra of the mixture at different concentration of 3-methylpyrazole.

Deconvolution in the case of signal overlap. In addition to lower sensitivity, benchtop NMR spectrometers also suffer from decreased spectral resolution compared to high field instruments. This leads to appreciable signal overlap in mixtures, as is illustrated in this work. Figure S6 shows that in cases of partial overlap, deconvolution methods may be used to obtain representative peak-integrals. A mixture at micromolar concentration of the compounds shown in S3 was measured, with the addition of acetonitrile (also at 40 μM), which leads to partial signal overlap of acetonitrile (2.01 ppm) and the investigated 3-methylpyrazole (2.25 ppm). Reference deconvolution using the FIDDLE algorithm is applied so that the two signals are baseline separated, and direct integration is possible. Also in this case of moderate overlap, a linear response of signal to the increase in concentration is observed, as indicated in S6.

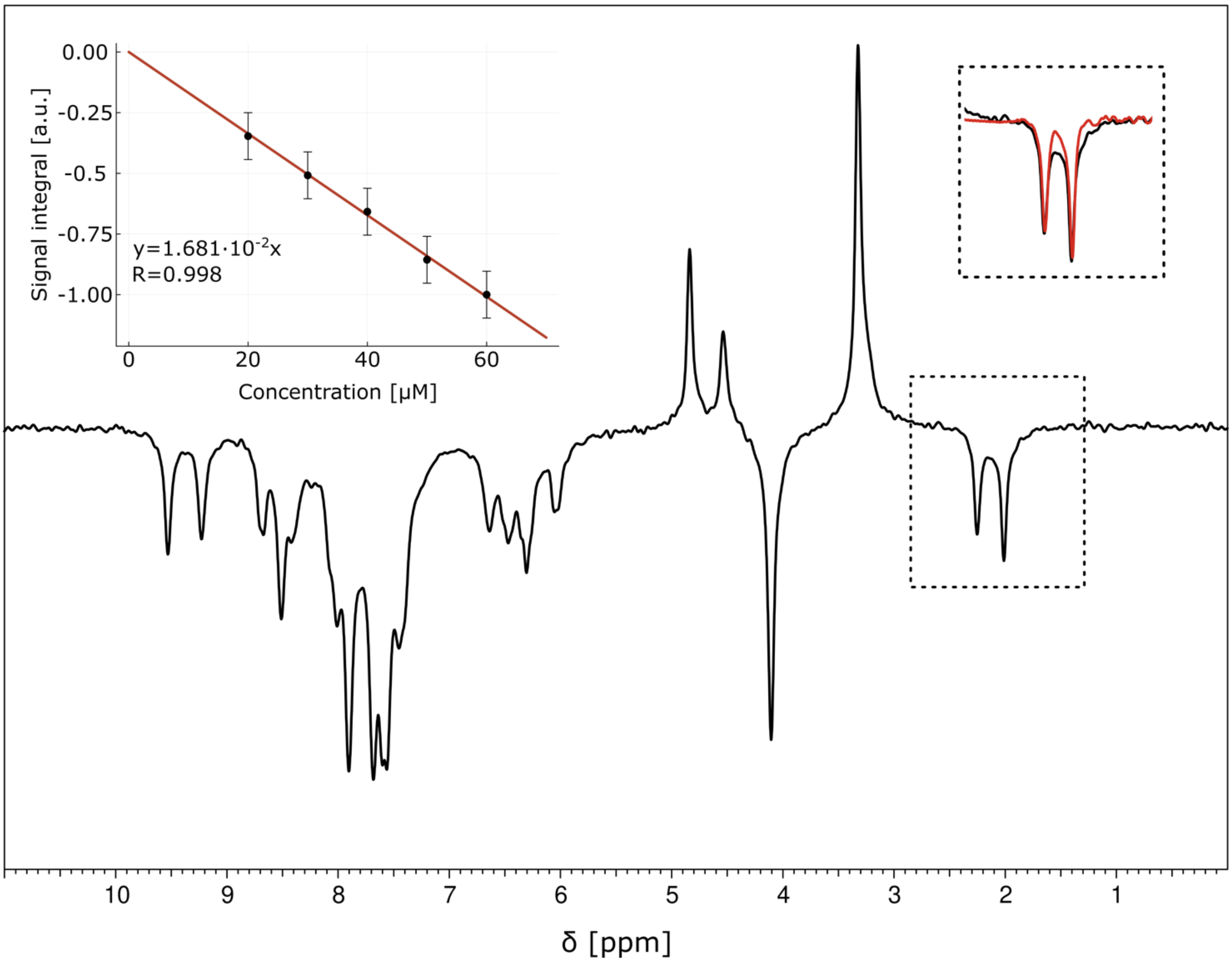

Figure S6. SABRE-enhanced ^{1}H NMR spectra of a mixture at micromolar concentration. The methyl signals of 3-methylpyrazole and acetonitrile are enclosed in the dashed box. Insert left: linear dependence of methyl signal of 3-methylpyrazole *versus* concentration (after deconvolution). Insert right: methyl signals of 3-methylpyrazole and acetonitrile before (black) and after (red) reference deconvolution. Error bars correspond to the estimated inter-sample variation (see main text).

REFERENCES

1. Kelly Iii, R. A.; Clavier, H.; Giudice, S.; Scott, N. M.; Stevens, E. D.; Bordner, J.; Samardjiev, I.; Hoff, C. D.; Cavallo, L.; Nolan, S. P., Determination of N-heterocyclic carbene (NHC) steric and electronic parameters using the [(NHC) Ir (CO) 2Cl] system. *Organometallics* **2008,** *27* (2), 202-210.

2. Ellermann, F.; Saul, P.; Hövener, J.-B.; Pravdivtsev, A. N., Modern manufacturing enables magnetic field cycling experiments and parahydrogen-induced hyperpolarization with a benchtop NMR. *Analytical Chemistry* **2023,** *95* (15), 6244-6252.
3. Yang, J.; Xin, R.; Lehmkuhl, S.; Korvink, J. G.; Brandner, J. J., Development of a fully automated workstation for conducting routine SABRE hyperpolarization. *Scientific Reports* **2024,** *14* (1), 21022.
4. Richardson, P. M.; Parrott, A. J.; Semenova, O.; Nordon, A.; Duckett, S. B.; Halse, M. E., SABRE hyperpolarization enables high-sensitivity 1 H and 13 C benchtop NMR spectroscopy. *Analyst* **2018,** *143* (14), 3442-3450.
5. Delaglio, F.; Grzesiek, S.; Vuister, G. W.; Zhu, G.; Pfeifer, J.; Bax, A., NMRPipe: a multidimensional spectral processing system based on UNIX pipes. *Journal of biomolecular NMR* **1995,** *6*, 277-293.
6. Morris, G. A., Compensation of instrumental imperfections by deconvolution using an internal reference signal. *Journal of Magnetic Resonance (1969)* **1988,** *80* (3), 547-552.
